# COVID-19 and smoking: an association requiring urgent attention

**DOI:** 10.1017/jsc.2020.26

**Published:** 2020-09-21

**Authors:** Vinoth Kumar Kalidoss, Satvinder Singh Bakshi

**Affiliations:** 1 Department of Community and Family Medicine, AIIMS Mangalagiri, Post box – 522503, Guntur, Andhra Pradesh, India; 2 Department of ENT and Head & Neck Surgery, AIIMS Mangalagiri, Post box – 522503, Guntur, Andhra Pradesh, India

To the editor,

The COVID-19 pandemic has emerged as the greatest challenge to the healthcare system worldwide. The pathogenesis involves the infection and replication of the virus in the epithelial cells of the respiratory system leading to severe acute respiratory syndrome and death. As on 25th April 2020 more than 187,000 deaths have been reported worldwide and the number of people infected stands at 2,724,809 (World Health Organization, 2020). Smoking is directly linked to the development of chronic pulmonary disease and smokers are vulnerable to many respiratory viruses. Besides, smoking is a risk factor for the development of many conditions such as cancer and cardiovascular disease, the presence of these conditions increases the morbidity and mortality in patients with COVID-19 (Emami, Javanmardi, Pirbonyeh, & Akbari, 2020). Some studies have already indicated that smoking has proven to worsen the prognosis and outcome in COVID-19 infections (Vardavas & Nikitara, 2020). The underlying mechanism may the reduced mucosal immunity and increased permeability of respiratory epithelial cells following chronic inflammation due to smoking. Another possible mechanism may be the increase in the expression of angiotensin-converting enzyme 2 receptor, which is also a binding receptor for the COVID-19 virus (Brake et al., 2020).

The enforcement of lockdowns, uncertainties about income, news regarding the pandemic and prolonged isolation measures will increase the psychological stress on patients which may, in turn, lead to an increase in the smoking habit (Patwardhan, 2020). Also ongoing de-addiction programs may also suffer a setback.

This scenario calls for an urgent increase in the anti-tobacco campaign. We as healthcare professionals must counsel smokers and prevent the emergence of new smokers. A clear message should be sent to patients that although these are stressful times, smoking is not a solution. Proper guidance to patients who smoke regarding the delirious effects of smoking especially concerning the COVID-19 pandemic should be imparted. They should be educated in terms of prevention of both active and passive smoking and given counseling on the availability of de-addiction services, coping with mechanisms for withdrawal symptoms which include balanced diet, regular physical activity, adequate sleep and nicotine replacements such as nicotine gums. Patients can be directed to a host of education material and motivational videos available on the internet. In addition, telemedicine services can be used by patients to communicate with their treating physicians and virtual group support sessions organized for psychological support and motivation. Opportunistic advice to patients on relapse prevention and to watch for increased smoking tendencies can be given. Training sessions can be organized for healthcare professionals in terms of the WHO 5A's model for tobacco cessation incorporating components such as ask, advice, assess, assist and arrange.

In summary, smoking is possibly a modifying factor for the aggravation of COVID-19. There is increased psychological stress which may lead to an increase in smoking during the pandemic. It is vital that healthcare providers recognize this disturbing trend and take active measures to prevent the rise in the number of smokers.
